# Adrenal insufficiency in patients with Prader-Willi syndrome

**DOI:** 10.3389/fendo.2022.1021704

**Published:** 2022-11-17

**Authors:** Marcin Jerzy Kusz, Aneta Monika Gawlik

**Affiliations:** ^1^ Department of Pediatrics and Pediatric Endocrinology, Medical University of Silesia, Katowice, Poland; ^2^ The Faculty of Medical Sciences, The Doctoral School of the Medical University of Silesia, Katowice, Poland

**Keywords:** PWS, Prader-Willi syndrome, adrenal insufficiency, Synacthen, LDSST

## Abstract

The generalized dysfunction of the hypothalamic-pituitary axis in patients with Prader-Willi syndrome (PWS) is the most likely cause of hypogonadism, inadequate growth hormone secretion, excessive appetite and associated obesity, impaired body temperature regulation, and hypothyroidism. The syndrome is also related to an increased risk of central adrenal insufficiency, although its prevalence remains unknown. The results of the studies in which different methods of pharmacological stimulation were used do not provide conclusive outcomes. As a result, there are no clear guidelines with regard to diagnosis, prevention, or long-term care when adrenal insufficiency is suspected in patients with PWS. Currently, most patients with PWS are treated with recombinant human growth hormone (rhGH). It has been confirmed that rhGH therapy has a positive effect on growth, body composition, body mass index (BMI), and potentially on psychomotor development in children with PWS. Additionally, rhGH may reduce the conversion of cortisone to cortisol through inhibition of 11β-hydroxysteroid dehydrogenase type 1. However, its influence on basal adrenal function and adrenal stress response remains unexplained in children with PWS. This paper reviews the literature related to the hypothalamic-pituitary-adrenal axis dysfunction in the PWS patient population with a focus on children.

## Introduction

Prader-Willi syndrome (PWS), which was first described in 1956 (1), is caused by the loss of paternally expressed imprinted genes at chromosome 15q11-q13. One in 10000-30000 live-born neonates is diagnosed with this condition. Due to the phenomenon of genomic imprinting, genes located in this region are expressed only on the paternally-derived chromosome, while the same genes on the maternally-derived chromosome undergo methylation and are not expressed and therefore are silenced. Deletion of a fragment of the paternal chromosome (60% of cases) is the most common mechanism for the loss of expression, followed by disomy of the maternal chromosome (35%) and disruption of the imprinting process, which rarely occurs (up to 5%) (2). The loss of expression of the paternally-derived genes leads to the occurrence of PWS. DNA methylation testing can detect 99% of cases (3).

Many physical, psychological and behavioral complications are associated with the course of the disease. Clinical manifestations are age-dependent (4), but not gender-dependent. In addition, the syndrome is the leading genetic cause of childhood obesity. Life expectancy depends on the age of diagnosis, the initiation of multi-specialty medical care and appropriate treatment, as well as the occurrence, course, and severity of complications. However, if the weight is well regulated, patients with PWS can reach the seventh decade (5).

The generalized dysfunction of the hypothalamic-pituitary axis is the likely basis for many symptoms and complications associated with PWS. In the life-threatening context, the hypothalamic-pituitary-adrenal (HPA) axis is of crucial importance. Symptoms of central adrenal insufficiency (CAI) are mainly due to glucocorticoid deficiency, which may manifest as reduced weight gain or prolonged jaundice in newborns. General weakness, recurrent infections, headache, muscle and joint pain are reported in older children. In the case of partial glucocorticoid deficiency, the disease may be asymptomatic and manifest only in stressful situations. Underestimation and underdiagnosis of CAI in the context of PWS patients (and children in particular) may lead to severe consequences. Temperature instability is common, with a tendency toward lower body temperature when compared to general population. Fever may be absent even during severe infection (2). This, combined with a decreased cortisol response in stressful situations in CAI patients, can lead to recurrent and life-threatening infections, often with a need for hospitalization, especially since it has been reported that up to 44% of death cases in PWS are due to respiratory tract infections (6). On the other hand, it is well known that glucocorticoid excess, which may be caused by chronic supplementation with glucocorticoids in cases of CAI overdiagnosis, may lead to decreased bone mineral density, osteoporosis, weight gain, hypertension, or immunosuppression. Additionally, it may also lead to hyperglycemia with insulin resistance, which may also be potentially enhanced by the above-mentioned growth hormone treatment therapy. The effect of isolated hypothalamic or pituitary dysfunction on the production of mineralocorticoids, including aldosterone, is limited, and electrolyte disturbances are usually not reported (6).

The diagnosis of CAI is based on the determination of adrenocorticotropic hormone (ACTH) and cortisol levels in the morning. CAI can be suspected when the cortisol level is low (< 83 nmol/L), the ACTH level is normal or decreased, and the electrolyte level is normal. An HPA-axis stimulation test is crucial for establishing the diagnosis. In the context of PWS patients, the choice of the test depends on the experience and capabilities of the research center and the availability of various stimulation methods, e. g. metyrapone is not available in the US at all. It requires overnight hospitalization and increases the risk of adrenal crisis, while the insulin tolerance test (ITT) is related to the risk of significant hypoglycemia, especially in children at risk such as PWS patients. Given the considerable safety profile, corticotropin analogs such as tetracosactrin (Synacthen^®^), which is a synthetic ACTH, are recommended as first-line stimulation tests, especially the low-dose tetracosactrin stimulation test (LDSST) (1 µg), which is usually very well tolerated (7).

The aim of this study was to review the literature related to the HPA axis disorders in the context of improving the quality of medical care for PWS patients. The aim of the authors was to provide up-to-date information on the prevalence and risks of CAI in PWS patients with a focus on children. Recommendations for CAI screening in PWS children are provided together with the dosage in hydrocortisone replacement therapy when it is detected. The authors believe this may lead to a better understanding of the presented issue among endocrinologists and general practitioners and to a better medical care of PWS patients, especially during illness or when hospitalization is indicated.

## Materials and methods

The literature review was based on the available papers published in the PubMed database between 1963 and June 2021. The search criteria used in the Medical Subject Headings (MeSH) were as follows: “Prader-Willi” [All Fields] AND (“adrenal glands”[MeSH Terms] OR (“adrenal”[All Fields] AND “glands”[All Fields]) OR “adrenal glands”[All Fields] OR “adrenals”[All Fields] OR “adrenalitis”[All Fields] OR “adrenally”[All Fields] OR “epinephrine”[MeSH Terms] OR “epinephrine”[All Fields] OR “adrenal” [All Fields])”.

Only original papers in English were analyzed. Case reports or literature reviews were not included in the review.

## Results/analysis of selected papers

Based on the criteria, 65 potentially relevant papers were identified. After analyzing the titles, abstracts and whole papers, ten articles (8–17) that met the criteria were included in the detailed analysis. The excluded papers included systematic reviews, case reports and papers that did not provide adequate insight regarding the prevalence, diagnosis or treatment of CAI.

The ten studies performed between 2008 and 2020 presented the results of dynamic tests which were performed to assess the risk of CAI. A total of 445 patients with PWS were enrolled, including 244 children. The studies consisted in the use of stimulation tests and the consecutive assessment of ACTH and/or cortisol secretion. The Synacthen test was used in 5 studies, the metyrapone test was used in 3 studies, the insulin test was used in 3 studies and the glucagon test (GT) in a single study. In the case of an abnormal test result, a confirmatory test was performed in 3 studies (the Synacthen test was used twice, while the metyrapone test was used once). **Table 1** shows cortisol and ACTH levels, the diagnostic criteria adopted by the investigators in the study protocol and the number of patients diagnosed with CAI in a given study. In total, CAI was diagnosed in 38 (8.5%) patients, including 33 children. In this group, the metyrapone test was used in 29 patients, the Synacthen test was used in eight subjects, while the ITT was used in a single patient. Information about the protocols of the stimulation tests with the conclusions drawn by the investigators is given in **Table 1**.

**Table 1:** Summary of the protocols and the results of the stimulation tests used by the investigators.

Study (author; year; citation)	Patients(n; department; age)	Morning cortisol level prior to testing	Method(s) (test/dose)	Level considered normal (min cut-off)	Diagnosis of CAI	Conclusions	Authors’ further comments
R. F. De Lind Van Wijngaarden et al., 2008 (12)	25 children hospitalized in the ICU	Median value of157.2 nmol/l (52.4–446.9) considered normal by the authors	A single metyrapone test (30 mg/kg)	ACTH ≥ 33 pmol/l at 0730 h	15 (60%)	A high prevalence of CAI in PWS may explain cases of sudden deaths, especially during infection.	CAI can develop over time. Therefore, a single normal test result does not rule out the occurrence of CAI in the future. Further studies are warranted.
R. F. De Lind Van Wijngaarden et al., 2009 (15)	20 children hospitalized in the ICU	Not assessed	A single metyrapone test (30 mg/kg)	ACTH ≥ 33 pmol/l at 0730 h	13 (65%)	Increased risk of the sleep apnea syndrome in PWS, especially in diagnosed CAI.	Changes in respiratory parameters can be even greater in stressful situations than during stimulation tests.
O. Nyunt et al., 2010 (10)	41 children	Median value of 223 ( ± 116) nmol/l considered normal by the authors	Synacthen test(1 µg)	Cortisol > 500 nmol/l measured at 30 min	0	Results contrary to the above studies; unstable level of ACTH in the blood sample which is subject to greater changes compared to cortisol.	It would be necessary to extend the diagnosis of CAI by performing the metyrapone test or the insulin-induced hypoglycemia test.
S. Farholt et al., 2011 (11)	65 patients (including 18 children < 17 years of age)	Median value of 194 (58-1020) nmol/l considered normal by the authors	Synacthen test in 57 patients (0.25 mg/m^2^) and the insulin tolerance test in 8 patients	Cortisol > 500 nmol/l measured at 30 min or increase in cortisol of ≥ 250 nmol/l	0	CAI rare in PWS in adults and children. Routine prophylactic treatment with hydrocortisone should be performed only if clinically indicated.	No significant gender effect on cortisol levels during stimulation.
A. Corrias et al., 2011 (8)	84 children	Median value of 341.7 ( ± 172.8) nmol/l considered normal by the authors	First, Synacthen test (1 µg), followed by the confirmatory Synacthen test (0.25 mg/m^2^).	Cortisol > 500 nmol/l measured at 30 min or increase in cortisol of ≥ 250 nmol/l.	4 (4.8%)	Recommended tests: LDSST and the assessment of morning cortisol and ACTH levels in all children with PWS as the element of the primary diagnosis of the function of the HPA axis.	In the case of normal stimulation tests, a repeat diagnosis should be performed periodically. However, further studies are warranted to determine the frequency of diagnostic procedures.
G. Grugni et al., 2013 (9)	53 adult patients	Median value of 336.6 ( ± 140.7) nmol/l considered normal by the authors	First, Synacthen test (1 µg), followed by the confirmatory Synacthen test (0.25 mg/m^2^)	Cortisol ≥ 500 nmol/l measured at 30 min	4 (7.5%)	Maintaining the above recommendations. A regular repeat diagnosis every 2 - 4 years is recommended.	Cooperation between centers and the development of diagnostic standards for CAI in PWS are essential to obtain more reliable data.
V. Beauloye et al., 2015 (17)	20 children	Considered normal by the authors; exact numerical values were not provided	The insulin tolerance test in 6 patients, glucagon test in 13 patients, both tests in a single patient	Cortisol > 550 nmol/l measured at 30 min or increase in cortisol of > 250 nmol/l	1 (5%)	CAI is rare in PWS.Increased risk of the sleep apnea syndrome in PWS was not confirmed.	The study has methodological limitations due to its retrospective nature and multicenter data collection.
Kathryn S. Obrynba et al., 2017 (14)	21 patients aged 4-53 years	Not assessed	Synacthen test (1 µg/m^2^, max. 1 µg) followed by the metyrapone test (30 mg/kg, max 3g)	Synacthen test: cortisol ≥ 427.6 nmol/l;The metyrapone test:11-deoxycortisol ≥ 200 nmol/l at 0800 h, regardless of the cortisol level	0	Clinically significant CAI is rare in PWS.The metyrapone test is a preferred method for the diagnosis of CAI in PWS.	Metyrapone is poorly available.The test requires close observation to monitor for potential adverse effects.
Yuji Oto et al., 2018 (16)	36 children	Median value of496.6 (391.7 – 653) nmol/l considered normal by the authors	Insulin tolerance test(0.1 IU/kg)	Morning level of ACTH 1.1-11 pmol/land cortisol > 165.6 nmol/lPeak (stimulation) ACTH level > 11 pmol/l and peak cortisol > 499.3 nmol/l or increase in cortisol by ≥ 9.1 µg/dl (251 nmol/l)	0	Delayed peak cortisol level in PWS compared to the controls -probably due to the hypothalamic dysfunction.	Further studies are warranted to confirm the findings.
AGW Rosenberg et al., 2020 (13)	82 adult patients	Median value of 325.5 (126.0 – 764.0) for the metyrapone test;median value of 172.5 (93.0 – 545.0) nmol/l, 185.5 (175.0 – 265.0) nmol/l, 233.0 (119.0 – 502.0) nmol/l, 229.0 (102.0 – 384.0) nmol/l for the ITT, depending on the center; all values considered normal by the authors	The multiple-dose metyrapone test (750 mg) or the insulin tolerance test (0.15 IU/kg)	Metyrapone test - 11-deoxycortisol > 230 nmol/l;insulin test – cortisol > 500 nmol/l, > 450 nmol/l for the British patients	1 (1.2%)	CAI is rare in adults with PWS. Routine treatment with hydrocortisone is not recommended in stressful situations or perioperatively.	Only 28% of patients were treated chronically with rhGH. Growth hormone deficiency can potentially mask adrenal insufficiency.

## Discussion

De Lind van Wijngaarden et al. performed one of the first studies on CAI in 2008. Overnight single-dose metyrapone tests were performed in randomly selected 25 children who were in a pediatric intensive care unit. Because metyrapone inhibits cortisol synthesis, it causes an increased demand for ACTH production, a situation mimicking stress. Based on the above and the fact that salivary cortisol levels were considered normal, de Lind van Wijngaarden et al. suggested that CAI became apparent only during stressful conditions when an increased demand for cortisol occurred. Salivary cortisol levels were found not to be useful in identifying PWS patients at risk of CAI. All patients with PWS should be treated with hydrocortisone during stressful situations if it is not possible to exclude CAI using the metyrapone test (12).

A similar study was conducted one year later. It was based on the assessment of the prevalence and severity of the sleep apnea syndrome during sleep. Poorer results were found, especially in children with CAI, which could confirm a common cause of the abnormality in the form of hypothalamic dysfunction. The authors found no significant differences in ACTH or cortisol levels between children who were later diagnosed with CAI and those who were not diagnosed with it (15).

In turn, in 2010, Nyunt et al. conducted their study on a group of 41 children with PWS. Following Synacthen administration, a normal response to stimulation was reported in all children. According to Nyunt et al., a high percentage of patients with CAI in a study conducted by de Lind van Wijngaarden et al. could be explained by adopting the level of ACTH, which is an unstable compound, as a criterion for the diagnosis of CAI. It would be necessary to extend the diagnosis of CAI by performing an overnight metyrapone test or the insulin-induced hypoglycemia test (10).

In 2011, Corrias et al. conducted a study on 84 children with PWS. In the first phase, decreased cortisol levels were found in 12 patients as a result of the low-dose tetracosactrin stimulation test (LDSST). This test was repeated in one patient. The test with a standard dose was performed in nine patients. Finally, CAI was confirmed in 4 patients. Corrias et al. recommended the LDSST and the assessment of morning cortisol and ACTH levels as the element of the primary diagnosis of the function of the HPA axis in children with PWS. The morning cortisol level was found to be of poor diagnostic value in diagnosing CAI even though basal cortisol levels were lower in the CAI group, but in both groups the values largely overlapped. Corrias et al. did not provide clear cut-off values indicating the need for further investigation with a LDSST. Substitution treatment with hydrocortisone seems to be warranted in moderate and severe stressful situations in all subjects with PWS who were not tested or were diagnosed with CAI (8).

In 2013, Grugni et al. conducted a study on 53 adults with PWS. The research plan was similar to the one in the above study. In the first phase, decreased cortisol levels were obtained after stimulation in eight patients. The tests were repeated and four patients showed persistent suboptimal responses. Grugni et al. stressed the recommendation to test each PWS patient for CAI. However, according to them, caution should be employed about establishing a diagnosis of CAI based solely on a single stimulation test. Once a decreased cortisol level is obtained using the low-dose short Synacthen test (LDSST) and in the case of no contraindications, the insulin-induced hypoglycemia test or the metyrapone test should be confirmatory. It is also possible to repeat LDSST or perform tests using the standard dose of Synacthen. Grugni et al. speculated that if LDSST had been performed in all patients with the basal cortisol cut-off of <250 nmol/l, no patient with CAI would have been missed. However, basal cortisol is overall a poor predictor of potential CAI (9).

In 2011, Farholt et al. performed a study in which all patients had a normal response to stimulation. Sixty-five patients with PWS were examined using the standard Synacthen test or the ITT. As opposed to de Lind van Wijngaarden et al., Farholt et al. were of the opinion that routine prophylactic treatment with hydrocortisone during acute illness in PWS was of concern due to many adverse effects such as obesity and hypertension. They recommended such treatment if it was clinically indicated. They found no significant gender effect on cortisol levels during stimulation (11).

In 2015, V. Beauloye et al., retrospectively reviewed cortisol response in 20 PWS children following either an ITT or a GT. Only a single patient showed insufficient cortisol response after ITT. Basal cortisol levels did not differ significantly between children with PWS and the controls. An overnight polysomnographic study was also conducted, but the results did not support the hypothesis of an increased risk of the sleep apnea syndrome in PWS as suggested by R. F. De Lind Van Wijngaarden et al. The link between CAI and the cases of sudden deaths in PWS was considered unlikely (15, 17).

In 2017, Obrynba et al. conducted a study on 21 patients with PWS. All patients underwent the Synacthen test, followed by the metyrapone. A third of patients who underwent the Synacthen test had peak cortisol below the level considered normal. However, one subject of the 21 patients had an inconclusive result of an overnight metyrapone test. This patient had normal stimulation test results in the first test. The authors found no difference in mean peak cortisol during the LDSST between patients treated with GH and those who did not undergo such treatment. According to Obrynba et al., the Synacthen test may lead to overdiagnosis of CAI, and the metyrapone test is the preferred diagnostic method. However, it is not widely available and requires close observation to monitor for potential adverse effects of transient adrenal insufficiency (14).

In 2018, Oto et al. conducted a study on 36 PWS children using ITT. None of the subjects was diagnosed with CAI. However, two-thirds showed delayed peak cortisol levels compared to the controls. According to Oto et al., the delay related to the response to ITT may signify the existence of a central obstacle in adjustment of the HPA axis of unknown origin and is related to latent CAI. Higher basal cortisol was observed in PWS patients when compared to control children. However, the difference was insignificant. Further research is required on the possible relationship between the altered pattern of cortisol secretion and hypoglycemia and cases of sudden death in PWS (16).

In their most recent study, Rosenberg et al. (2020) (13) investigated 82 adult patients with PWS. The multiple-dose metyrapone test was given to 46 subjects. Thirty-six patients underwent ITT. None of the patients underwent two tests. An abnormal response indicating CAI was found in one patient. Rosenberg et al. stressed that in the study population only 28% of subjects were treated chronically with rhGH. While growth hormone deficiency can potentially mask adrenal insufficiency, CAI is almost non-existent in adult PWS patients.

As presented, the authors of the above studies did not provide clear morning cortisol cut-offs to perform stimulation tests. These tests were performed in patients with basal cortisol considered normal by the authors. As mentioned before, basal cortisol is of limited value in detecting potential CAI cases because of its low sensitivity and specificity.

The analysis of stimulation tests shows that a high percentage of CAI in PWS patients was found by de Lind van Wijngaarden et al. (28 out of 38, 73.6%), most of whom (33; 86%) were children. There could be an increased annual sudden death rate in PWS patients with non-detected CAI and more severe cases do not make it to adulthood. However, the percentage of CAI cases in PWS subjects was much lower in research performed later. Four studies involving a total of 163 PWS patients, including 95 children, found that CAI was not diagnosed in any subject (10, 11, 14, 16). The differences in the results may be due to the type of a stimulation test and different diagnostic criteria. As mentioned above, the criteria for the diagnosis of CAI adopted by de Lind van Wijngaarden et al. were based on the concentration of ACTH, which is highly unstable after collection due to proteolytic degradation (18). In addition, there are some doubts about the ACTH concentration accepted as normal. According to some authors, the adopted cut-off point is too high, which leads to overdiagnosis of CAI (10, 13). Furthermore, according to Rosenberg et al., a single-dose metyrapone test has limited diagnostic usefulness due to the short suppression time of the HPA axis, which does not give sufficient time for the increase in adrenal steroid production. The metyrapone test was also carried out by Obrynba et al. in 2017. The study also included children, but CAI was not diagnosed in any patient. However, 11-deoxycortisol levels were used to determine cut-off values for CAI diagnosis (14). If we compare the studies of Corrias et al. (2011) and Grugni et al. (2013) on two different groups (children and adults, respectively) CAI was diagnosed more frequently in adults than in children (7.5% vs 4.8%) (8, 9). It is unclear whether the differences are related to the age of the patients or the adopted stimulation test and cut-off points.

Early detection of CAI is crucial due to the need for the rapid introduction of hydrocortisone replacement therapy. As mentioned earlier, glucocorticoid deficiency can be associated with many adverse effects and complications, which may not be found in non-stressful situations. Furthermore, many authors have suggested that some deaths could potentially be related to undetected CAI.

Due to the differences in the study results, no clear guidelines have been developed for the diagnosis, management, or long-term care of patients with PWS or CAI. Some centers inform patients with PWS of the possibility of adrenal insufficiency and recommend low-dose hydrocortisone treatment in the home setting when the infection starts or exacerbates (19). However, Heksch et al. recommended screening tests for CAI before major surgery and glucocorticoid replacement of 30-50 mg/m^2^/day dived three times daily during mild or moderate insufficiency, and 75-100 mg/m^2^/dose given immediately prior to surgery or anesthesia. However, the authors did not recommend specific tests and reported that the selection of the optimal test remained unclear (20). The insulin test is considered the gold standard for diagnosing adrenal insufficiency (21). However, it is related to the risk of significant hypoglycemia, especially in children at risk. In turn, the metyrapone test increases the risk of adrenal crisis (8). Furthermore, some studies suggest a high number of false-positive results when the Synacthen test is used, although it is considered an alternative to the insulin test (14). The type of genetic defect does not seem to affect the risk of CAI (12).

There is also an important issue of different cortisol assays used in laboratories. Newer cortisol assays have much improved specificity, and cortisol values are approximately 20% lower when compared to previous assays. Lowering the cortisol response threshold of 500 nmol/l by 20% for CAI detection has been suggested. However, it requires further research. Significant variability is observed depending on the type of cortisol assay used. Therefore, it is recommended to check the reference ranges with the laboratory (22). Since the exact assays used worldwide are currently not standardized as regards CAI, it is still unclear which exact cut-off values should be adopted.

**Table 2** includes all stimulation tests that are currently used in the diagnosis of CAI. LDSST is presented as in the Endocrine Society guidelines. Different interpretation values depend on the laboratory assay used (6, 23).

**Table 2:** All stimulation tests used to diagnose central adrenal insufficiency in children (6, 22).

Test	Advantages	Disadvantages	Interpretation of the results
LDSST 1 ug synthetic ACTH/Synacthen	- relatively easily available and simple to perform - quite high sensitivity, an acceptable equivalent of the insulin test - high safety profile	- a potentially high number of a false positive diagnosis of CAI - low specificity, a negative result does not rule out CAI	Cortisol level > 500 nmol/l measured at 30 min rules out the diagnosis of CAI (23)
Insulin test 0.05 – 0.15 U/kg	- high sensitivity and a high diagnostic value - considered the gold standard in the diagnosis of adrenal insufficiency	- the risk of significant hypoglycemia, especially in children at risk - poorly tolerated by patients - necessary experience to perform the test	Cortisol level > 500 (≥ 550 (6)) nmol/l rules out the diagnosis of CAI (23)
Metyrapone test 30 mg/kg	- easy to perform - high sensitivity	- increases the risk of adrenal crisis - limited availability (unavailable in the USA) leads to the lack of standardization - according to some authors, a single test is of limited diagnostic value	ACTH level >17 pmol/l (23) and/or 11-deoxycortisol level >200 nmol/l rules out the diagnosis of CAI (6, 23)

Based on the literature review, primary adrenal insufficiency (PAI) has not been reported in PWS patients. It should be suspected in case of decreased basal cortisol levels with elevated ACTH levels. Standard dose Synacthen stimulation test (SDSST) with 250 µg of tetracosactrin is recommended to confirm the diagnosis (7). Cortisol value of ≥ 500 nmol/l at 0, 30 or 60 min. excludes PAI.

## Conclusions

In the 10 studies, CAI was diagnosed in 38 (8.5%) patients, including 33 children. The metyrapone test was performed in 29 subjects, LDSST in 8 patients and ITT in one subject (8, 9, 12, 13, 15, 17). After reviewing the papers included in this literature review and after a detailed review of the advantages and disadvantages of specific stimulation tests, it may seem reasonable to exclude CAI in each patient with PWS due to the likely increased risk of the HPA axis dysfunction, obligatorily when the symptoms that may indicate it are present (**Table 3**) (7). As mentioned before, it has been speculated that if LDSST had been performed in all patients with the basal cortisol cut-off of <250 nmol/l, no patient with CAI would have been missed (9). However, this value is quite high as it includes cortisol values that are within the normal serum cortisol range. With the newer cortisol assays we recommend mandatory CAI exclusion by stimulation tests in patients with the basal cortisol cut-off of < 138 nmol/l (5 µg/dL). Basal morning cortisol should be assessed regularly every 3 – 6 months in PWS patients as soon as diagnosis is made. Patients should be closely monitored for CAI symptoms [**Table 3**] and in case of their occurrence, even despite normal basal cortisol levels, CAI should be excluded by performing stimulation tests.

**Table 3:** Symptoms of adrenal insufficiency in children which indicate the need for the early assessment of hypothalamic-pituitary-adrenal axis function (7).

Clinical symptoms	Laboratory symptoms
- abnormal weight gain, weight loss - vomiting - recurrent infections - excessive fatigue - muscle and joint pain - headache and dizziness	- hypoglycemia - hyponatremia - normokalemia (CAI) - hyperkalemia (chronic kidney disease) - anemia - lymphocytosis - eosinophilia

LDSST is the proposed first-line stimulation test since it is simple to perform and is characterized by a low risk of adverse effects. When the test result does not exclude CAI (cortisol level < 500 nmol/L at 30 min.), the insulin test would be the optimal choice to confirm the diagnosis. It is possible provided the center has adequate experience in its use and there are no clinical contraindications for performing such a test (cardiovascular/neurological conditions, untreated hypothyroidism, glycogen storage disease). The metyrapone test or a repeat LDSST is an alternative to the insulin test.

Currently, most patients with PWS are treated with rhGH and there is no consensus on the need for HPA axis testing before starting the rhGH therapy (24). Such therapy has a positive effect on growth, body composition, BMI, and potentially on psychomotor development in children with the syndrome (25). Additionally, rhGH may reduce the conversion of cortisone to cortisol through inhibition of 11β-hydroxysteroid dehydrogenase type 1. However, the influence of therapy with rhGH on basal adrenal function and adrenal stress response remains unexplained in children with PWS (26). No statistically significant differences were demonstrated between the prevalence of CAI in patients treated with rhGH and those who were not on such therapy (14). In addition, no effect of rhGH therapy was observed on the increased mortality rate in patients with PWS (27), which is estimated at 3% annually (28), and its most common cause is related to exacerbation of respiratory infections (29). In addition, post-mortem studies found below-average-sized adrenal glands in some children, which could indicate undiagnosed subclinical adrenal insufficiency (30).

Hypogonadotropic hypogonadism is typical of PWS patients due to the hypothalamic dysfunction. Adrenarche is clinically defined as the start of pubarche or axillarche with other symptoms such as change in the body odor or acne. Premature adrenarche, which occurs in about 14 - 30% of PWS patients, also indicates HPA dysfunction, but rarely progresses to precocious puberty, although such cases were reported in both sexes (31, 32). It is possible that premature adrenarche is yet another clinical feature of PWS not necessarily connected with hypogonadotropic hypogonadism as their timings do not seem to be correlated (32). Based on this speculation, it seems unlikely that CAI prevalence is correlated with premature adrenarche or pubarche.

- It is recommended that the hydrocortisone dose is increased for up to 3 days then decreased back to the basic replacement dose, but the exact length of increased hydrocortisone dose is dependent on the patient’s clinical state.

In children with PWS, it seems reasonable to perform the diagnosis of CAI at least once. It should be repeated if symptoms occur. The proposed hydrocortisone replacement doses are given in **Table 4** (7, 20). At present, however, there are no universal recommendations on the necessity, method, or frequency of conducting/repeating the diagnostic procedures for CAI in PWS. Additionally, there are no guidelines for replacement. Further research is warranted to develop a generally accepted diagnostic and therapeutic plan. The prevalence and the mechanism of occurrence of CAI in children with PWS are still unexplained. To date, there have been no specific diagnostic criteria for CAI in PWS, and the opinions of different authors about its prevalence are inconclusive.

**Table 4:** Proposed replacement doses of hydrocortisone in children in central adrenal insufficiency, depending on the stressful situation, with the authors’ modification (7, 20).

Situation	Daily dose
Basic replacement	7.5 – 15 mg/m^2^ in 2 - 4 divided doses
Infection/stress	30 – 50 mg/m^2^ in 3 divided doses (20) OR: 2 – 3 times the basic replacement dose*
Surgery, patients in a more severe condition	2 mg/kg before surgery, then following depending on the weight of the patient: - ≤ 10 kg - 25 mg/24h - > 10-20 kg - 50 mg/24h - > 20 kg - 100 mg/24h prepubertal or 150mg/24h pubertal (7) OR: 0 – 3 yrs: bolus of 25 mg + 25 mg/24h in 3 divided doses 3 – 12 yrs: bolus of 50 mg + 50 mg/24h in 3 divided doses > 12 yrs: bolus of 100 mg + 100 mg/24h in 3 divided doses*

## Author contributions

MK identified potential papers, analyzed selected papers, and took the lead in writing the manuscript. AG supervised the project, assessed the performed analysis and recommended changes, contributed to the final version of the manuscript. All authors contributed to the article and approved the submitted version.

## Acknowledgments

The authors wish to thank Assistant Professor Arkadiusz Badziński, DHSc, a medical translator and interpreter for translating the manuscript.

## Conflict of interest

The authors declare that the research was conducted in the absence of any commercial or financial relationships that could be construed as a potential conflict of interest.

## Publisher’s note

All claims expressed in this article are solely those of the authors and do not necessarily represent those of their affiliated organizations, or those of the publisher, the editors and the reviewers. Any product that may be evaluated in this article, or claim that may be made by its manufacturer, is not guaranteed or endorsed by the publisher.
